# Multi-beam X-ray ptychography for high-throughput coherent diffraction imaging

**DOI:** 10.1038/s41598-020-76412-8

**Published:** 2020-11-11

**Authors:** Yudong Yao, Yi Jiang, Jeffrey A. Klug, Michael Wojcik, Evan R. Maxey, Nicholas S. Sirica, Christian Roehrig, Zhonghou Cai, Stefan Vogt, Barry Lai, Junjing Deng

**Affiliations:** 1 Advanced Photon Source, Argonne National Laboratory, Lemont, IL 60439 USA; 2 Center for Integrated Nanotechnologies, Los Alamos National Laboratory, Los Alamos, NM 87545 USA

**Keywords:** Imaging techniques, Microscopy

## Abstract

X-ray ptychography is a rapidly developing coherent diffraction imaging technique that provides nanoscale resolution on extended field-of-view. However, the requirement of coherence and the scanning mechanism limit the throughput of ptychographic imaging. In this paper, we propose X-ray ptychography using multiple illuminations instead of single illumination in conventional ptychography. Multiple locations of the sample are simultaneously imaged by spatially separated X-ray beams, therefore, the obtained field-of-view in one scan can be enlarged by a factor equal to the number of illuminations. We have demonstrated this technique experimentally using two X-ray beams focused by a house-made Fresnel zone plate array. Two areas of the object and corresponding double illuminations were successfully reconstructed from diffraction patterns acquired in one scan, with image quality similar with those obtained by conventional single-beam ptychography in sequence. Multi-beam ptychography approach increases the imaging speed, providing an efficient way for high-resolution imaging of large extended specimens.

## Introduction

X-ray ptychography is an emerging coherent lensless imaging technique that can produce a high-resolution image of an extended sample^1–3^. It has become popular in many research areas (e.g., materials science^4–7^, biology^8–11^, electronics^12–14^, and optics^15–17^) because quantitative information can be obtained on both sample and illumination, with a high-spatial resolution not limited by optics but rather by the maximum angle of scattered signals. Recent advances in technique and algorithm developments also made ptychography more robust to deal with many problems, such as partial coherence^18^, scanning errors^19,20^, and beam variation^21^.

Ptychography doesn’t put a limit on the imaging field-of-view (FOV), but the increase of FOV comes at the cost of long scanning time and large data volume. To reduce the experiment time, one can use a higher-flux illumination achieved by opening the source aperture. This method is effectively used in some incoherent imaging systems such as scanning transmission X-ray microscopy (STXM)^22^, however, it contradicts the coherence requirement in ptychography due to the decrease of spatial coherence. Recent efforts were made on reconstruction algorithms to deal with partial coherence so that one can use a source with higher flux. For example, the development of the mixed-state method greatly relaxes the coherent requirement of ptychography and enables the use of partial coherence illumination to significantly reduce the exposure time by delivering higher flux on the sample^18,23^. In practical scans, a continuous motion scheme (called “fly-scan”)^24–26^ has been recently implemented in ptychography to speed up imaging by avoiding mechanical overheads, while the decoherence effect caused by the continuous motion is solved in the reconstruction.

Imaging with multiple illuminations while maintaining enough coherence degree on each illumination is also a novel and effective approach for high-throughput imaging since separated sample sites with each illumination are imaged simultaneously. This idea was firstly demonstrated in visible light domain using either mutually incoherent beams (via spectrally^27^ or spatially^28^ separated) or mutually coherent beams^29^. In the second case, the interference of exit surface waves from two separated sources makes reconstruction complicated, and careful processing on the data is required to filter out the interference terms^29^.

In this paper, we proposed and demonstrated this multi-beam approach for X-ray ptychography, which was completed independently from another similar work^30^ published recently. In the experiment, two focused beams were produced by a Fresnel zone plate (FZP) array, and used for ptychography scanning. The imaged FOV was doubled in this demonstration while leaving the scan time and acquired data volume unchanged. We also incorporated the partial coherent illumination and the fly-scan technique to the multi-beam approach to further increase the ptychographic throughput. The transmission function of both sample and probe for two illumination beams were reconstructed simultaneously, and the two sample regions were successfully separated and had similar image quality compared with the conventional single beam ptychography.

## Methods

For multi-beam ptychography, the object *O*(*r*) is scanned by multiple illuminations $P_m(r)$ called the “probe” ($m = 1, 2,\ldots , M, M$ is the total number of beams), which can be generated by several sets of focusing optics or a pinhole array. When the separation distance between neighbouring apertures that define the probes is larger than the transverse coherence length of X-ray beam in this plane, those multiple beams can be assumed to be mutually incoherent. For the *m*th illumination, the complex-valued exit wave from the object at the *j*th scan point can be expressed as

1 $$\begin{aligned} \psi _m(r, j) = O_m(r-r_j) P_m(r), \end{aligned}$$

where $O_m$ denotes a patch of object illuminated by the corresponding illumination beam $P_m$ while different parts of the object are imaged simultaneously by multiple probes, *r* is the coordinate on object plane and $r_j$ is the *j*th ($j = 1,2,\ldots ,J, J$ is the total number of scan points) scan position. The expected diffraction intensity collected by the detector is the superposition of the diffraction intensities from multiple illumination beams,

2 $$\begin{aligned} \begin{aligned} I(k,j)&= \sum _m |\mathscr {P}_m \{\Psi _m(k, j) \}|^2 \\&= \sum _m| \mathscr {P}_m \left\{ \mathscr {F} \{O_m(r-r_j) P_m(r) \} \right\} |^2, \end{aligned} \end{aligned}$$

where *k* is the reciprocal coordinate with respect to the real space coordinate *r*, $\mathscr {F}$ represents the Fourier transform operator with the detector situated at far-field. The distance among the diffraction patterns on the detector from multiple beams is determined by the beam spacing and $\mathscr {P}_m$ is the operator that shifts the diffraction pattern corresponding to the *m*th beam on the detector plane.

In the real experiment, we need to consider various disadvantageous experimental effects that reduces the coherent property of the X-ray beam, such as beam and/or sample fluctuations^31^, partial coherence illumination light source^18^ and fly-scan data collection strategy^24–26^. To overcome these decoherence effects, a mixed-state decomposition approach was recently developed^18^ by including mutually incoherent probe modes in the reconstruction. In the multi-beam ptychography method, extra orthogonal states $P_{m,n}$ are introduced to each illumination beam during the ptychographic reconstruction. Consequently, the recorded far-field intensity measurement is the incoherent summation of the diffraction intensity from these orthogonal probe modes of each illumination beam,

3 $$\begin{aligned} \begin{aligned} I(k,j)&= \sum _m \sum _n| \mathscr {P}_m \{\Psi _{m,n}(k, j) \}|^2 \\&= \sum _m \sum _n | \mathscr {P}_m \left\{ \mathscr {F} \{O_m(r-r_j) P_{m,n}(r) \} \right\} |^2. \end{aligned} \end{aligned}$$

The propagated exit wave $\Psi '_{m,n}(k,j)$ on the detector plane can be updated by the following method,

4 $$\begin{aligned} \Psi '_{m,n}(k,j) = \mathscr {P}^{-1}_m \left\{ \frac{\sqrt{I^E(k,j)}}{\sqrt{I(k,j)}} \right\} \Psi _{m,n}(k,j), \end{aligned}$$

where $$I^E(k,j) $$ represents the experimental recorded diffraction intensity, and $$\mathscr {P}_m^{-1}$$ is the operator that shifts the diffraction pattern for the *m*th beam back to the center. Propagation of the updated solution $$\Psi '_{m,n}(k,j)$$ back to real space provides an updated exit wave $$\psi '_{m,n}(r,j)$$ estimate. Finally, the optimal exit wave is decomposed into the complex transmission function $$O_m(r)$$ and the probe function $$P_{m,n}(r)$$ and the optimization is solved by the least-squares maximum likelihood solver^32^. In the meantime, the sample scan position is refined during the iterative reconstruction procedure.

## Experiment and results

**Figure 1:**
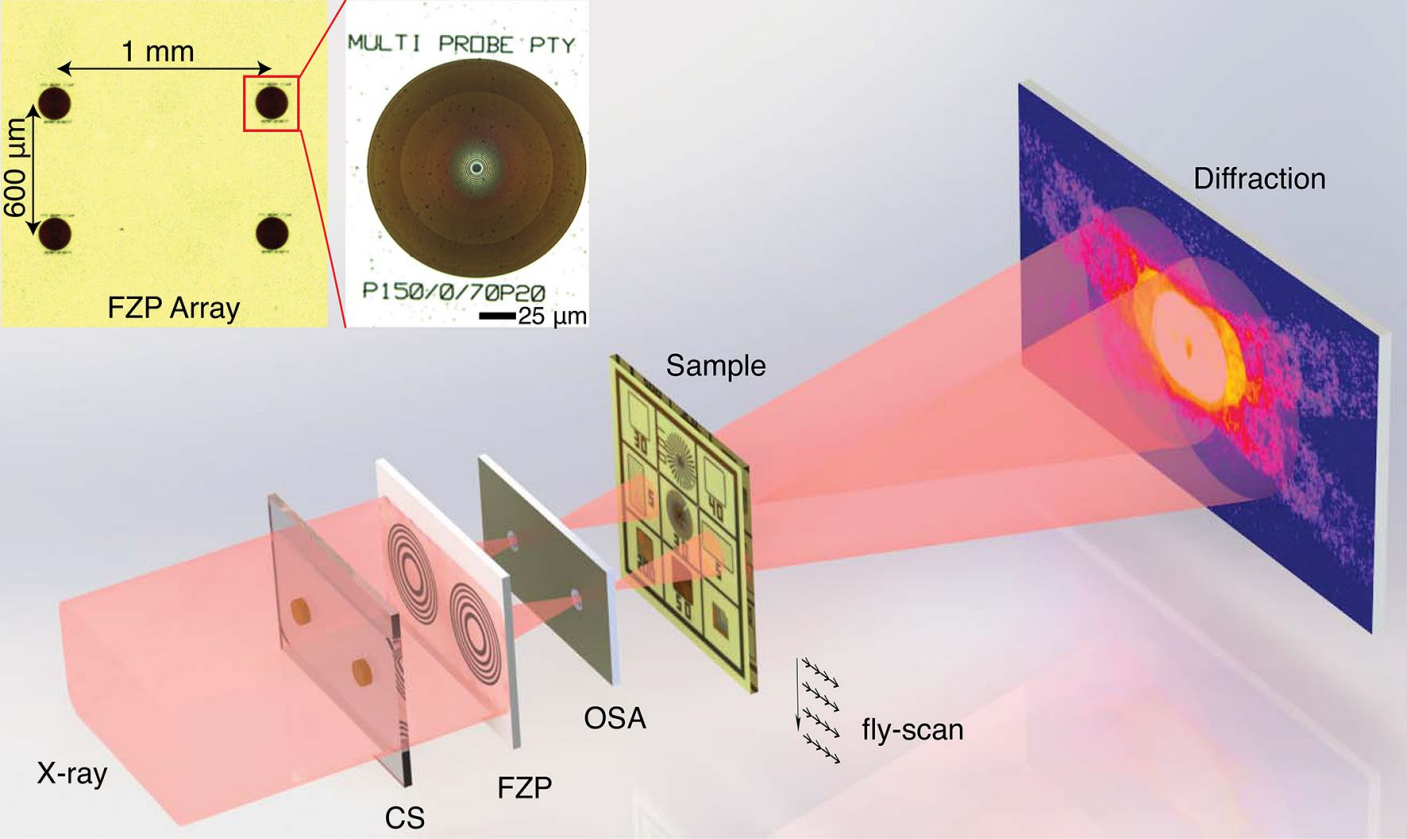
Schematic of multi-beam ptychography. Multiple illuminations are generated by a set of focusing optics comprised of Fresnel zone plates (FZP), central stops (CS) and order sorting apertures (OSA). The inset images are $5\times$ and $40\times$ visible light micrographs of the house-made FZP array with 150 $\upmu$m diameter and 70 nm outmost zone width.

We demonstrated this technique on a fluorescence microscope at beamline 2-ID-D at the Advanced Photon Source (APS) in Argonne National Laboratory. Ptychography experiment was performed at 8.8 keV photon energy defined by a double-crystal Si(111) monochromator. Gold Fresnel zone plate arrays with 2 (row) $\times$ 4 (column) were fabricated on a Si$_3$N$_4$ membrane with a thickness of 700 nm. Each zone plate has a diameter of 150 $\upmu$m and 70 nm outermost zone width, giving the focal length of 74.52 mm at 8.8 keV X-ray energy. The X-ray flux for each focused beam is $\sim 5 \times 10^8$ photons/s. The inset of Fig. 1 shows the horizontal interval between zone plates is 1 mm, and 600 $\upmu$m in the vertical direction. Figure 1 shows the schematic of the experimental setup: two zone plates on the FZP array were chosen to focus the incoming X-ray beam; in order to obtain only the first-order focusing, central beam stops (CSs) and order sorting apertures (OSAs) with the same spacing as the two zone plates were installed upstream and downstream of the FZP array. Two gold CSs with 60 $\upmu$m diameter and 80 $\upmu$m height were fabricated on 2 mm $\times$ 2 mm silicon-nitride membranes with 500 $\upmu$m off center. The OSA array was fabricated by Oxford Lasers, Inc. with customized specifications: holes with a diameter of 30 $\upmu$m were made on 300 $\upmu$m-thick tungsten plate using laser micro-drilling, with hole spacing the same as that on the FZP array. During the alignment, the two CSs were mounted on two sets of motorized positioners driven by picomotor actuators and then were aligned with the FZPs, respectively. The OSA array was mounted on a rotation stage sitting on the top of three-axis translation stages. After moving the OSA array into the beam, the rotation stage combined with translation stages were used to align two OSAs to the selected FZPs. The sample was placed about 500 $\upmu$m downstream of the focal plane, forming two illuminations with a spot size of about 1 $\upmu$m on two regions of the sample. The sample was raster-fly-scanned in a rectangular pattern with x axis as fly-scan direction^25^ (z is X-ray direction). X, y encoder positions from the sample translation stages were used as the initial positions for ptychography reconstructions and position correction algorithm^32^ was implemented during the reconstruction process. The far-field diffraction patterns were collected by a Dectris Eiger 500K detector (75 $\upmu$m pixel size) placed 1.75 m downstream of the sample.

**Figure 2:**
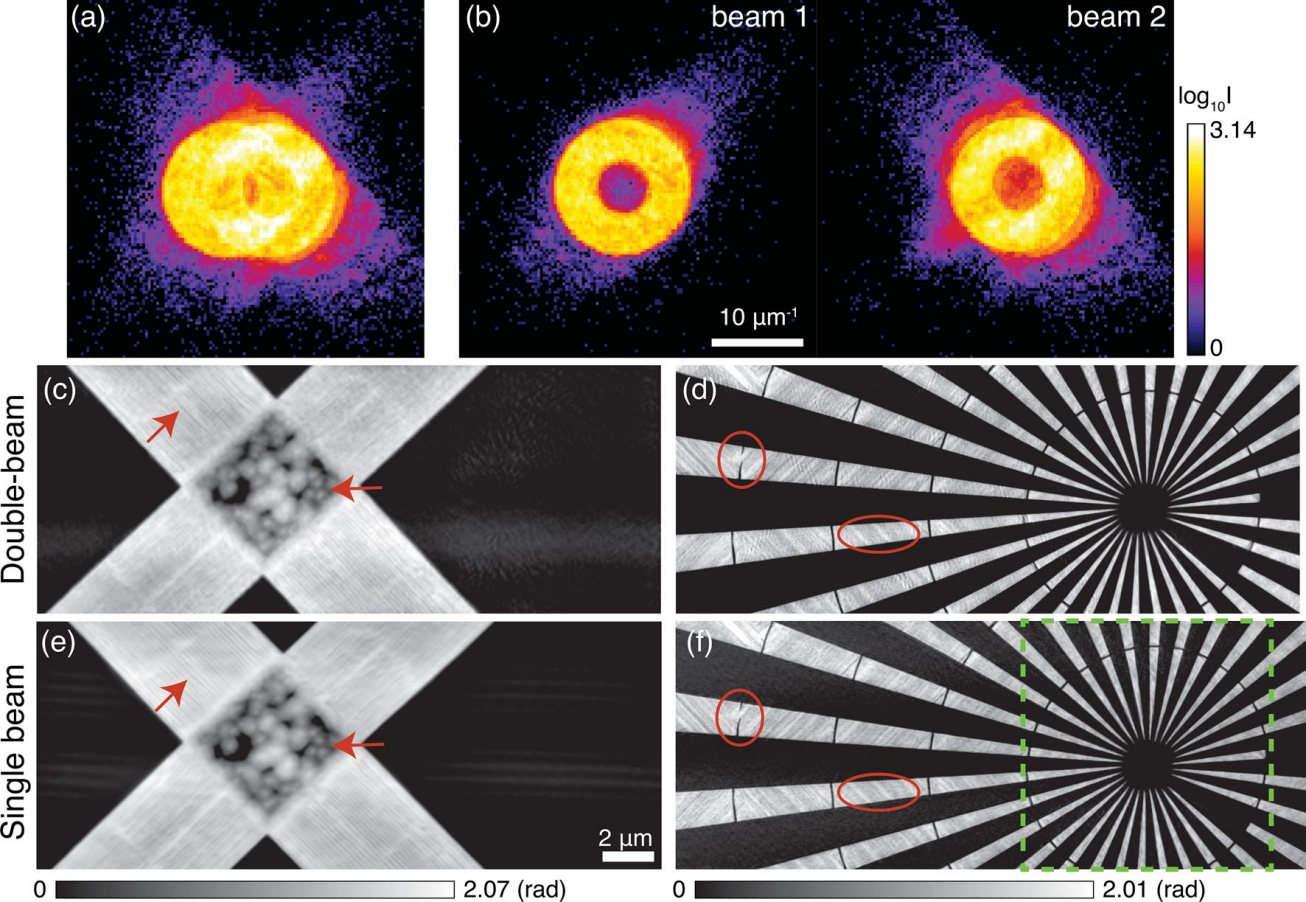
Double-beam ptychography with two illuminations focused by two zone plates with a separation of 1 mm. Measured diffraction patterns with (**a**) double beams and (**b**) each single beam (beam 1 and 2). (**c**) and (**d**) are reconstructed phase images of double-beam ptychography showing the two sample regions that were simultaneously scanned. Two single-beam ptychographic scans were taken in sequence by blocking one of the two beams at a time, their reconstructed results are shown in (**e**) with beam 1 and in (**f**) with beam 2, respectively.

In the first experimental demonstration, two zone plates with a horizontal spacing of 1 mm as shown in the inset of Fig. 1 were chosen to produce two illuminations. The horizontal transverse coherence length on the FZP plane was defined by upstream white beam slits of 80 $\upmu$m horizontal width at 44 m distance, giving a coherence length of about 77 $\upmu$m. In this case, the zone plate with $150\, \upmu \hbox {m}$ acceptance diameter was illuminated with partially coherent X-rays in the horizontal direction, while covered by fully coherent X-rays in the vertical direction($\sim \, 310\, \upmu \hbox {m}$ vertical transverse coherence length at FZP plane). Therefore, the mixed-state decomposition approach as mentioned in Eq. (3) is very important to deal with the partial coherence in the reconstruction. Meanwhile, the two illuminations focused by these two zone plates could be assumed to be mutually incoherent because the empty spacing of $850\, \upmu \hbox {m}$ between FZPs was much larger than the horizontal transverse coherence length. These two beams simultaneously illuminated on a resolution test sample (see Supplementary Fig. S1) which was composed of 500 nm thick Au patterns fabricated by electron beam lithography. Figure 2a is a diffraction pattern recorded with these two beams illuminating on the gold test sample. The 1 mm separation between two zone plates only gave about 13.3 pixels ($75\,\upmu \hbox {m}$ pixel size) on the detector plane, the primary beams (two donut-shape rings) and their diffraction by the sample were observed to be largely overlapped with each other in this diffraction pattern. By selecting one of the zone plates at a time with an upstream $250\, \upmu \hbox {m}$ pinhole, the diffraction patterns from each single zone plate were acquired with the same exposure time and displayed in Fig. 2b. One can observe that the scattering signals in the diffraction pattern shown in Fig. 2a is a superposition of the scattering signals formed by each single beam shown in Fig. 2b. Moreover, no interference fringes exist in the diffraction intensity for double-beam illumination, indicating that two beams are mutually incoherent. A fly-scan ptychography was conducted with these two beams on this gold sample with a 100 nm step size in both horizontal and vertical directions and an exposure time of 10 ms, resulting in $\sim$ 10 min data acquisition time covering a FOV of $25\, \upmu \hbox {m} \times 10 \,\upmu \hbox {m}$ for each beam. The data acquisition time can be further improved by using advanced fly-scan trajectory, such as snake-raster scan pattern and spiral-scan pattern^33^. In the reconstruction, both probe functions used an array size of 128 $\times$ 128 pixels, giving a 25.6 nm pixel size in the real space. Figure 2c,d shows reconstructed phase images of the two regions of the sample that were illuminated by the two beams, using 10 probe modes. The imaged region shown in Fig. 2c is a big cross structure with some fine lines and dot features clearly resolved (denoted by red arrows), while the Siemens star with finest spokes of 100 nm was successfully resolved in Fig. 2d. For comparison, two single-beam ptychography scans with the same scan parameters were performed in sequence by selecting only one zone plate at a time with an upstream $250\, \upmu \hbox {m}$ pinhole. The reconstructed phase images of those two scans were shown in Fig. 2e,f, respectively. The dots and fine lines denoted by red arrows in Fig. 2c given by double-beam ptychography are consistent with the features in the single-beam reconstruction (Fig. 2e). These lines or stripes together with those examples marked by the oval shapes in Fig. 2d,f were also observed in ptychography scans (see Supplementary Fig. S2 and Fig. 5 in the reference paper^34^) on different areas but on the same window acquired by a high-resolution ptychography instrument-the Velociprobe^33^. Therefore, the line patterns are real and are presumably due to the defects from the fabrication process, displaying the parallelogram (or trapezoid) filling scheme of electron beam lithography^35^.

**Figure 3:**
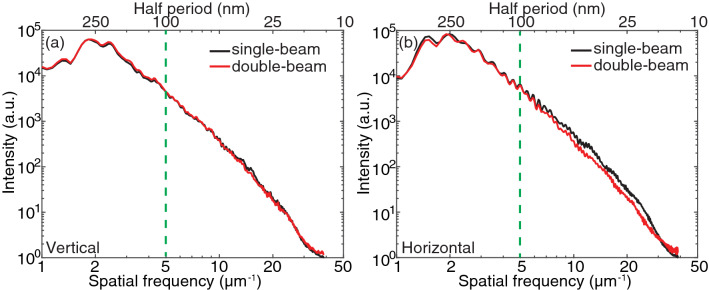
Power spectrum of the reconstructed region marked by the green box in Fig. 2f for double-beam and single-beam ptychography. (**a**) Vertical power spectrum density; (**b**) horizontal power spectrum density.

To access the quality and the spatial resolution of the double-beam ptychography method, the power spectral density (PSD)^25^ was calculated for the reconstructed region marked by the green box in Fig. 2f. The vertical and horizontal PSDs are shown in Fig. 3a,b, respectively. Both vertical and horizontal PSDs for the double-beam ptychographic reconstruction are consistent with the PSDs for single-beam reconstruction. The dashed green lines in Fig. 3 denote the size of the finest spokes (100 nm), showing that double-beam ptychography provides similar image quality for the smallest features compared with the single-beam ptychography in both vertical and horizontal directions. One can also observe that the horizontal PSD for double-beam ptychography shows a little degradation for high spatial frequency signals due to the largely overlapping of diffraction patterns in the horizontal direction. More importantly, ptychography with two beams has double acquisition efficiency and reduced data volume as the information from two sample regions are recorded in one scan.

**Figure 4:**
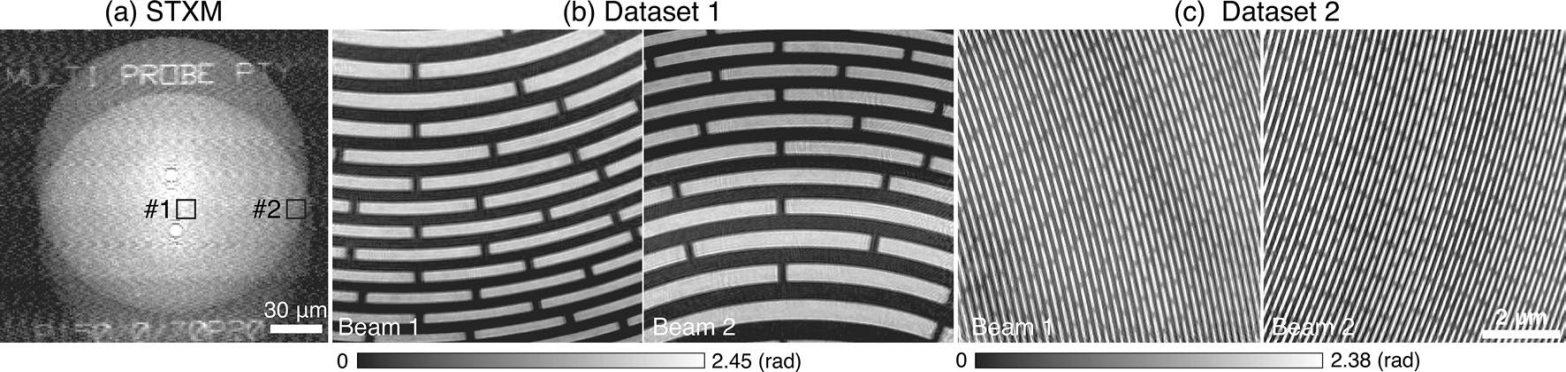
“Self-portrait” of the FZP array with double-beam ptychography. (**a**) STXM image of two zone plates with double-beam illumination. (**b**) and (**c**) Ptychographic reconstruction of two datasets extracted from two regions, marked by black boxes in (**a**), having different zone widths.

The two sample regions shown in Fig. 2c,d have very different structures with each other, which might help on the separation of the two object functions during the reconstruction. To explore the multiple beam method on sample regions with similar structures, an FZP array identical to the one used for the focusing optics was used as sample. A “self-portrait” scan with two beams was carried out on the FZP array with a scan region of $170\, \upmu \hbox {m}\times 180\,\upmu \hbox {m}$ for each beam, which took about 19 min with $1\, \upmu \hbox {m}$ step size. Figure 4a shows the STXM image, which was obtained by calculating the total integrated intensity of each diffraction pattern and then mapping the intensity to the corresponding scan point, revealing that the illuminated two zone plates were superposed in this image. Two sub-datasets with large and smaller zone features (marked in Fig. 4a) were extracted to do reconstruction, respectively. The ptychography data acquisition time for each sub-dataset was $\sim$5 min for $8\, \upmu \hbox {m}\times 8\,\upmu \hbox {m}$ single-beam FOV with a scan step size of 100 nm in both horizontal and vertical directions. In the reconstruction, probe size of 256 $\times$ 256 pixels was used, giving a 12.8 nm pixel size in the real space. Figure 4b shows the reconstructed phase images of the central area of the two zone plates, where large zone features of the FZP were imaged. The second dataset was from the outmost part of the FZPs. The small zones with a feature size of $\sim$ 70 nm on both zone plates were successfully reconstructed and well resolved as shown in the Fig. 4c. The successful reconstruction of the FZP shows that double-beam ptychography works well for two illuminated regions that have features with similar length-scale. It is worth mentioning that orientations of features in these two regions are different, as shown in Fig. 4c, which is helpful for the reconstruction algorithm to distinguish two imaged regions. To image samples with highly similar features or repetitive structures, wavefront modulations for illumination probes^30^ or additional constraints (e.g. total-variation regularization^36^) in the reconstruction algorithm may be helpful for separating different imaged regions.

**Figure 5:**
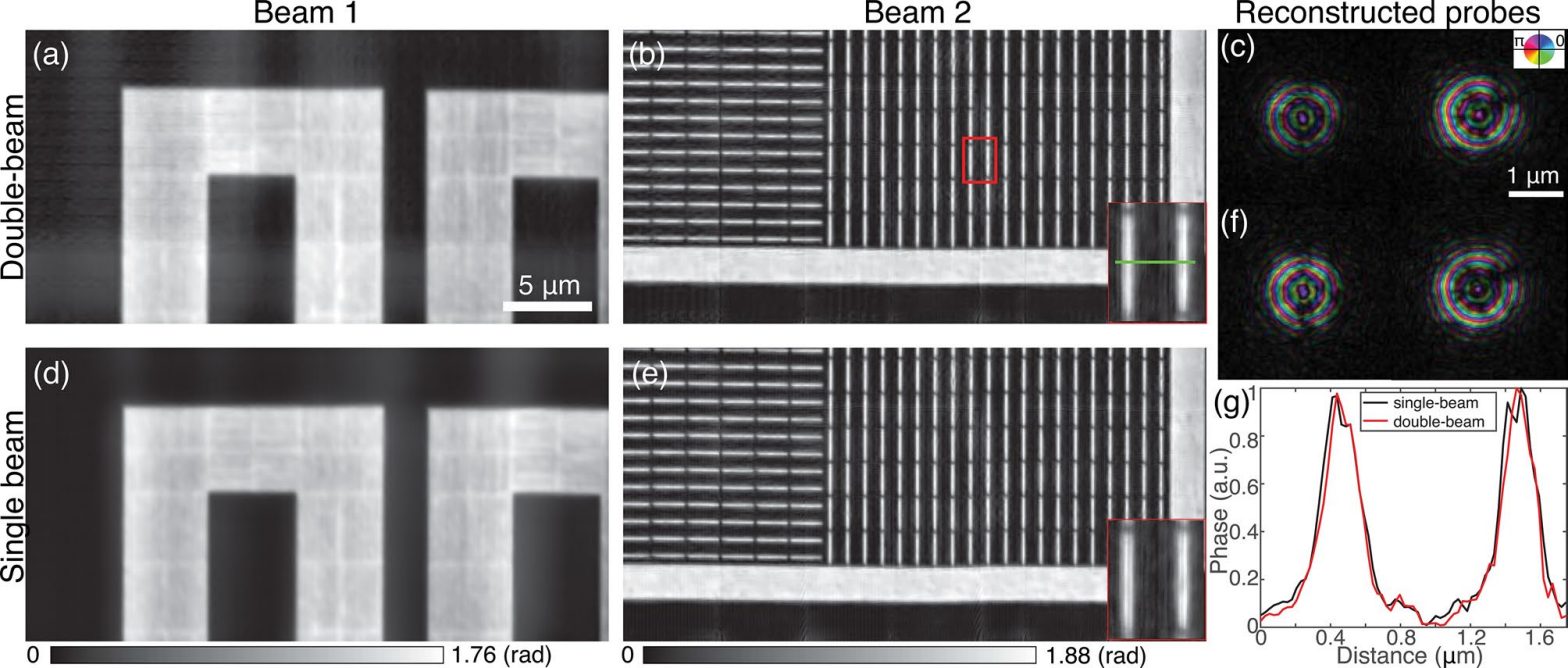
Reconstructions for double-beam ptychography with a beam separation of $600\, \upmu \hbox {m}$. Reconstructed phase images of two sample regions for (**a**) and (**b**) double-beam; (**d**) and (**e**) two single beams illuminating the same region, sequentially. Insets in (**b**) and (**e**) show a zoomed region denoted by the red box in (**b**). Reconstructed probes for (**c**) double-beam and (**f**) two single beams; a complex color scale is used with the brightness corresponding to amplitude and the hue indicating relative phase-shift. (**g**) Line-cut profile for the selected region marked by the green line in the inset of (**b**).

For the proof-of-concept experiment, a large separation of 1 mm between two zone plates was used in the above scans. To further increase the scan efficiency using multiple beams, one would consider decreasing the distance between the zone plates so that a higher number of beams can be used in one scan. To decrease the beam distance, we rotated the FZP array by 90$^\circ$ to obtain a spacing of $600\, \upmu \hbox {m}$ between zone plates. The decreasing separation between zone plates might cause interference between illuminations, which would bring difficulty in the reconstruction. The empty spacing between the two zone plates after rotation was $450\, \upmu \hbox {m}$ which is still larger than the horizontal transverse coherence length. Therefore, our proposed method should still work in this scenario. Double-beam ptychography with these two illuminations was performed on the gold test sample with a step size of 100 nm and 200 nm in the horizontal and vertical directions and an exposure time of 20 ms, respectively, yielding about 24 min data acquisition time. Figure 5a,b shows the two reconstructed regions (see the corresponding areas in Supplementary Fig. S1) that were illuminated by the two X-ray beams separated by $600\, \upmu \hbox {m}$, which were successfully separated and clearly visualized. The region in Fig. 5b shows the consistent pitch pattern with a line width of 200 nm. Again, for comparison, conventional single-beam ptychography scans were performed on these two areas in sequence, with their reconstruction results shown in Fig. 5d,e. The results with decreased separation between beams doesn’t show a loss in imaging quality. Compared with the results given by conventional single-beam ptychography, the double-beam method produces better image quality as demonstrated by the vertical bars shown in the inset in Fig. 5b, which are sharper than those shown in Fig. 5e. The line-cut profiles at the selected position (the green line in the inset of Fig. 5b) are given in Fig. 5g. It can be observed that the line-cut profile for single-beam ptychography shows unexpected structures induced by the position error, while the result for double-beam ptychography provides better image quality due to more constraints and better position refinement in the double-beam reconstruction. Ten probe modes were used to represent each beam in reconstructions. The main probe mode for these two beams are shown in Fig. 5c,f for double-beam and single-beam ptychography, respectively. It is worth mentioning that the two illumination beams in the double-beam ptychography experiment have different wavefronts, as shown in Fig. 5c, due to the imperfection in the FZP fabrication process. This nonsimilarity between two beams is helpful for separating two imaged regions, as well as the convergence of the reconstruction algorithm.

Further decrease in the beam separation is possible based on the above result with $600\, \upmu \hbox {m}$ separation. The smallest separation between our zone plates can be about $250\, \upmu \hbox {m}$ in the horizontal and $500\, \upmu \hbox {m}$ in the vertical (the empty spacing between zone plates larger than the traverse coherence length) in order to get mutually incoherent beams. Therefore, a 2 mm $\times$ 2 mm X-ray incident beam is able to accommodate an 8(H) $\times$ 4 (V) zone plate array. Considering the measured pixels are about two orders of magnitude more than the reconstructed pixels in the double-beam case, the oversampling criterion is still satisfied when the number of beams increases to 32, which in theory can improve the throughput of ptychography by 32 times. However, using more beams increases the complexity of the experiment especially for the optics alignment. As the proposed multiple-beam method in this paper assumes illuminations are mutually incoherent, it may fail when the interference between illuminations occurs as the beam separation continues to decrease. In this case, the interference among the beams need to be suppressed, for example, through the digital filtering of the acquired data^29^, or developing algorithms to deal with the partial coherence.

In addition to the data acquisition time, the reconstruction time is also an important factor that influences the ptychographic imaging efficiency. For the same FOV, the reconstruction time of double-beam ptychography is about the same with the time needed for two sequential single-beam ptychography reconstructions, while preserving the same image quality. The reconstruction speed for multi-beam ptychography can be further improved by algorithm development, such as implementing parallelization for multiple beams during the reconstruction process, thus further increasing the ptychography imaging efficiency.

## Conclusion

The work reported here demonstrates a multi-beam ptychography technique using zone plate focused beams. A set of focusing optics including a FZP array, central stops and OSA array was developed to focus the coming X-rays into multiple compact illuminations which were mutually incoherent. In this proof-of-concept experiment, two illuminations from the FZP array were used under several conditions to show the robustness of multiple-beam ptychography. All the objects and probes corresponding to multiple beams were successfully reconstructed, with imaging quality comparable with conventional single-beam ptychography. In some cases, multiple-beam ptychography works better in position correction because of more information constraints. Since different regions of the sample are illuminated simultaneously with multi-beams in one ptychographic scan, the imaged FOV is proportionally increased by a factor equal to the number of beams, while having a small data volume compared to sequential single-beam scans on the same FOV. Even though only two beams were demonstrated for this multiple-beam technique, more zone plates can be introduced by optimizing the beam separation and the transverse coherence length to further make use of the available X-ray flux to improve ptychography throughput.

## Supplementary information

Supplementary Figures.

## References

[1] Rodenburg J (2007). Hard-x-ray lensless imaging of extended objects. Phys. Rev. Lett..

[2] Thibault P (2008). High-resolution scanning x-ray diffraction microscopy. Science.

[3] Pfeiffer F (2018). X-ray ptychography. Nat. Photon..

[4] Holler M (2014). X-ray ptychographic computed tomography at 16 nm isotropic 3D resolution. Sci. Rep..

[5] Hruszkewycz SO (2012). Quantitative nanoscale imaging of lattice distortions in epitaxial semiconductor heterostructures using nanofocused x-ray bragg projection ptychography. Nano Lett..

[6] Shapiro DA (2014). Chemical composition mapping with nanometre resolution by soft X-ray microscopy. Nat. Photon..

[7] Donnelly C (2017). Three-dimensional magnetization structures revealed with x-ray vector nanotomography. Nature.

[8] Giewekemeyer K (2010). Quantitative biological imaging by ptychographic x-ray diffraction microscopy. Proc. Natl. Acad. Sci..

[9] Deng J (2015). Simultaneous cryo x-ray ptychographic and fluorescence microscopy of green algae. Proc. Natl. Acad. Sci..

[10] Diaz A (2015). Three-dimensional mass density mapping of cellular ultrastructure by ptychographic x-ray nanotomography. J. Struct. Biol..

[11] Deng J (2018). Correlative 3d x-ray fluorescence and ptychographic tomography of frozen-hydrated green algae. Sci. Adv..

[12] Guizar-Sicairos M (2014). High-throughput ptychography using Eiger: scanning X-ray nano-imaging of extended regions. Opt. Express.

[13] Deng J (2017). Nanoscale x-ray imaging of circuit features without wafer etching. Phys. Rev. B.

[14] Holler M (2017). High-resolution non-destructive three-dimensional imaging of integrated circuits. Nature.

[15] Kewish CM (2010). Ptychographic characterization of the wavefield in the focus of reflective hard x-ray optics. Ultramicroscopy.

[16] Schropp A (2010). Hard x-ray nanobeam characterization by coherent diffraction microscopy. Appl. Phys. Lett..

[17] Huang X (2013). 11 nm hard x-ray focus from a large-aperture multilayer laue lens. Sci. Rep..

[18] Thibault P, Menzel A (2013). Reconstructing state mixtures from diffraction measurements. Nature.

[19] Maiden AM, Humphry MJ, Sarahan MC, Kraus B, Rodenburg JM (2012). An annealing algorithm to correct positioning errors in ptychography. Ultramicroscopy.

[20] Zhang F (2013). Translation position determination in ptychographic coherent diffraction imaging. Opt. Express.

[21] Odstrcil M (2016). Ptychographic coherent diffractive imaging with orthogonal probe relaxation. Opt. Express.

[22] Rarback H, Schmahl G (1984). Recent results from the stony brook scanning microscope. X-Ray Microscopy.

[23] Enders B (2014). Ptychography with broad-bandwidth radiation. Appl. Phys. Lett..

[24] Pelz PM (2014). On-the-fly scans for x-ray ptychography. Appl. Phys. Lett..

[25] Deng J (2015). Continuous motion scan ptychography: characterization for increased speed in coherent x-ray imaging. Opt. Express.

[26] Huang X (2015). Fly-scan ptychography. Sci. Rep..

[27] Karl R (2015). Spatial, spectral, and polarization multiplexed ptychography. Optics Express.

[28] He X (2018). High-speed ptychographic imaging based on multiple-beam illumination. Opt. Express.

[29] Bevis C (2018). Multiple beam ptychography for large field-of-view, high throughput, quantitative phase contrast imaging. Ultramicroscopy.

[30] Hirose M, Higashino T, Ishiguro N, Takahashi Y (2020). Multibeam ptychography with synchrotron hard X-rays. Opt. Express.

[31] Clark J, Huang X, Harder R, Robinson I (2014). Dynamic imaging using ptychography. Phys. Rev. Lett..

[32] Odstrčil M, Menzel A, Guizar-Sicairos M (2018). Iterative least-squares solver for generalized maximum-likelihood ptychography. Opt. Express.

[33] Deng J (2019). The velociprobe: an ultrafast hard x-ray nanoprobe for high-resolution ptychographic imaging. Rev. Sci. Instrum..

[34] Deng, J. *et al.* Instrumentation and method developments of x-ray ptychography at the advanced photon source. In *X-Ray Nanoimaging: Instruments and Methods IV. Volume 11112 of Proceedings of SPIE* (ed. Lai, B.), 111120E (2019).

[35] Lu M, Tennant D, Jacobsen C (2006). Orientation dependence of linewidth variation in sub-50-nm Gaussian e-beam lithography and its correction. J. Vac. Sci. Technol. B..

[36] Chambolle A (2004). An algorithm for total variation minimization and applications. J. Math. Imaging Vis..

