# Supplementary material for: Multi-beam X-ray ptychography for high-throughput coherent diffraction imaging
Source: Sci Rep. 2020 Nov 11;10:19550. doi: 10.1038/s41598-020-76412-8 (PMC7658249; doi:10.1038/s41598-020-76412-8)
Supplement: Supplementary file 1 — Supplementary Figures. [file 41598_2020_76412_MOESM1_ESM.pdf]

# Multi-beam X-ray ptychography for high-throughput coherent diffraction imaging

Yudong Yao[1], Yi Jiang[1], Jeffrey A. Klug[1], Michael Wojcik[1], Evan R. Maxey[1], Nicholas S. Sirica[2], Christian Roehrig[1], Zhonghou Cai[1], Stefan Vogt[1], Barry Lai[1], and Junjing Deng[1,*]

[1] Advanced Photon Source, Argonne National Laboratory, Lemont, IL 60439, USA
[2] Center for Integrated Nanotechnologies, Los Alamos National Laboratory, Los Alamos, NM 87545, USA
[*] junjingdeng@anl.gov

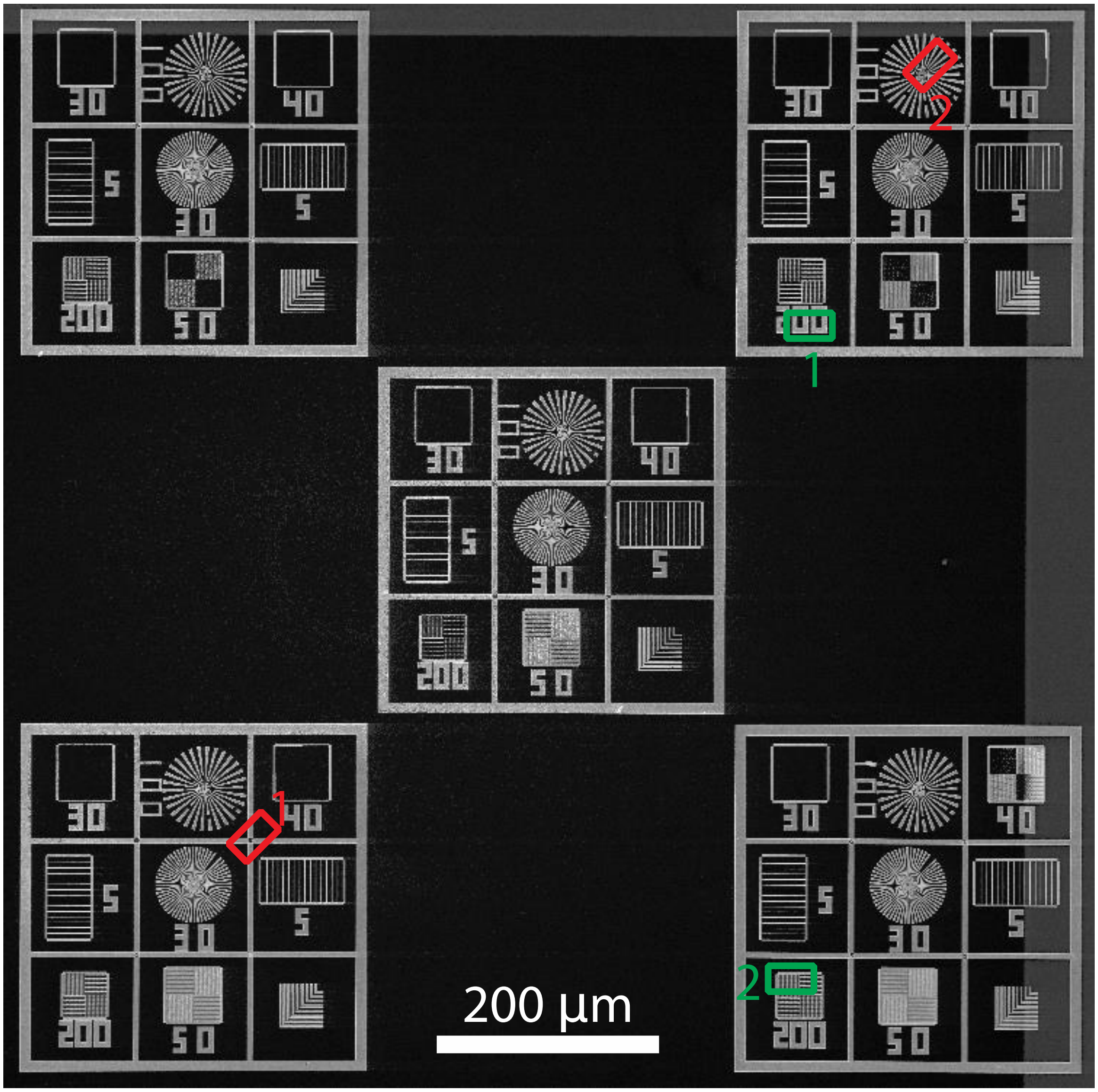


**Supplementary Figure S1.** Scanning electron microscopy (SEM) image for the Au test pattern sample on a 1 mm x 1 mm silicon-nitride window. In the double-beam ptychography with 1 mm-separation illuminations (Fig. 2), the sample was manually rotated by ~45 degrees, and two regions (marked by red boxes) with 1 mm separation were scanned. In the double-beam ptychography with 0.6 mm-separation illumination (Fig. 5), regions marked by the two rectangular boxes in green were scanned.

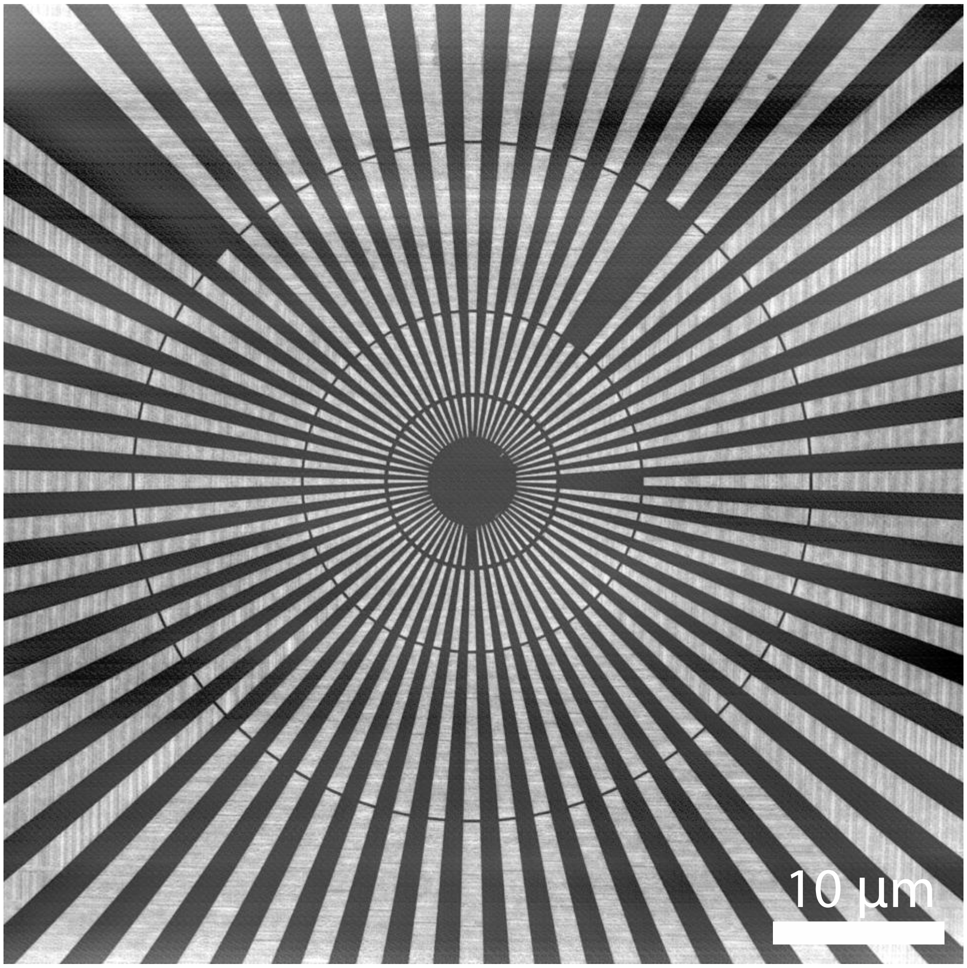


**Supplementary Figure S2.** Ptychographic image of a Siemens star with finest spokes of 30 nm on the same window obtained from the Velociprobe – a high-resolution X-ray nanoprobe which is able to provide sub-10 nm spatial resolution [33]. Visible lines or stripes are due to defects from the fabrication process.
